# The Editor's Letter-Box

**Published:** 1917-03-31

**Authors:** Sydney Pitt

**Affiliations:** 16 St. Andrew Street, Holborn Circus


**The Secret Compact? ! ! !**

THE MOUNTAIN IN LABOUR ONCE AGAIN.

[Copy of Correspondence.]

Royal British Nurses' Association and the College of Nursing, Ltd.

Dear Mr. Stanley,—I understand from Mr. Paterson that you have arranged with him that an undertaking shall be given with regard to the following points:—

(1) "The Registrar" is to be substituted for "the Secretary" in the draft Bill of the College for the registration of nurses.

(2) The Nursing Society of the Royal British Nurses' Association is to be carried on, and the private staff is to be continued on at least its present scale, with salaries and emoluments not less than those which they now receive.

(3) Miss Rundle is to be the Secretary of the Amalgamated Body.

(4) Miss Macdonald is to be the Registrar. While she continues to discharge her present duties she is to receive her present salary and emoluments, but in no event is her salary to be less than that of the Secretary.

(5) It is to be understood that one-third of the members of the first Council elected under the By-laws, and one-third of the members of the Amalgamated Body who may represent it on the Council under the proposed Bill for registration of nurses, shall be members of the Royal British Nurses' Association.

If you agree to the above, I shall be glad if you will kindly let me have an undertaking, signed by yourself and Sir Cooper Perry, that due effect shall be given to them.

I quite understand that you cannot legally bind the new Council, and that your acceptance can only be considered as an honourable undertaking that you will do your utmost to see that effect shall be given to these points, and that it must not be considered as involving any of you in any personal liability.—Believe me, yours truly,

(Signed) Sydney Pitt.

16 St. Andrew Street, Holborn Circus,

November 30, 1914.

